# Neutrophil‐to‐monocyte ratio is the better new inflammatory marker associated with rheumatoid arthritis activity

**DOI:** 10.1002/hsr2.1478

**Published:** 2023-08-02

**Authors:** Jamil M. A. S. Obaid, Malikah M. A. Almjydy, Maymouna A. Q. Garban, Fatima S. Q. Al‐hebari, Nusaibah A. H. Al‐washah

**Affiliations:** ^1^ Department of Medical Laboratory Sciences Faculty of Medicine and Health Sciences, Ibb University Ibb Yemen; ^2^ Department of Medical Microbiology Faculty of Science, Ibb University Ibb Yemen

**Keywords:** disease activity, inflammation, marker, neutrophil‐monocyte ratio, rheumatoid arthritis, sensitivity, specificity

## Abstract

**Background:**

Rheumatoid arthritis (RA) is a systemic autoimmune disease that chronically affects patients with episodes of inflammation. New inflammatory hematological markers were investigated for follow‐up, such as the neutrophil–monocyte ratio (NMR), lymphocyte monocyte ratio (LMR), and neutrophil–lymphocyte ratio (NLR). This study was conducted to determine the most useful marker based on studies of association with RA disease activity and correlation with the classical markers C‐reactive protein (CRP), erythrocyte sedimentation rate (ESR), and rheumatoid factor (RF).

**Methods:**

This case‐control study included 62 chronic RA patients who had previously been diagnosed and experienced episodes of symptoms while attending a variety of public and private rheumatology clinics in Ibb City, Republic of Yemen, for the period of September 1 to November 30, 2021. Twenty healthy volunteers were included in this study. Complete blood count, CRP, ESR, and RF levels were measured in all participants.

**Results:**

The total leukocyte count, neutrophil count, platelet count, NMR, LMR, and NLR were positively correlated with CRP and ESR, but the monocyte count was reversed. The area under the curve (AUC = 0.861, 95% confidence interval [CI] = 0.769–0.948) for the NMR cutoff value of 4.7 was equal to that of CRP and close to that of ESR. This NMR cutoff value had 87% sensitivity and 80% specificity. LMR and NLR cutoff values of 4.35 and 1.35, respectively, resulted in AUCs of (AUC = 0.807, 95% CI, 0.708–0.905) and (AUC = 0.699, 95% CI, 0.571–0.819); their sensitivity and specificity were 62.3%, 90%, 57.4%, and 80%, respectively.

**Conclusions:**

As a convenient and low‐cost inflammatory marker of RA activity, NMR outperformed LMR and NLR.

## 1 INTRODUCTION

Rheumatoid arthritis (RA) is a systemic autoimmune rheumatic disease that affects the synovium of joints, causing inflammation, bone damage, and finally disability.^1^ The cardinal pathogenic hallmark of RA is inflammation that manifests disease activity, Inflammation aggravates and subsides alternately, causing fluctuations in the RA disease course; therefore, rapid suppression of inflammation maximizes disease control.^2^ Thus, pro‐inflammatory cytokines such as interleukin (IL)‐1, IL‐6, IL‐15, IL‐18, and tumor necrosis factor‐α (TNF‐α), initiate changes represented by the characteristic signs of inflammation (redness, swelling, pain, and the perception of surface and internal heat). In contrast, some other cytokines, such as IL‐4 and IL‐10, act as anti‐inflammatory agents.^3^, ^4^ Pro‐inflammatory cytokines mediate the formation of inflammatory edema, which is responsible for the development of stiffness symptom.^5^

Potential prognostic markers for assessing RA disease activity had been investigated earlier, including the classical inflammatory markers, rheumatoid factors (RF), anti‐cyclic citrullinated peptide (anti‐CCP) antibodies, C‐reactive protein (CRP), and erythrocyte sedimentation rate (ESR) that are widely used as indicators of disease activity.^6^, ^7^ Other markers that had been investigated in RA patients and showed significantly higher serum levels in patients compared with healthy controls are high‐sensitivity CRP, IL‐6, TNF‐α, and IL‐10.^8^

Neutrophils, monocytes, lymphocytes, and platelets have been known to contribute to the development and progression of inflammation^9^ The neutrophil‐to‐monocyte ratio (NMR), lymphocyte‐to‐monocyte ratio (LMR), and neutrophil‐to‐lymphocyte ratio (NLR) have drawn attention in recent years as a novel, simple, and inexpensive inflammatory markers for many diseases, including RA.^10^, ^11^, ^12^, ^13^ Accordingly, this study was conducted to assess the most useful of the new markers NMR, LMR, and NLR based on their degree and significance of association with RA disease activity, and their correlation with the classical markers CRP, ESR, and RF.

## 2 MATERIALS AND METHODS

### 2.1 Subjects

This case–control study was conducted on 62 chronic RA patients previously diagnosed (not less than 1 year) and with episodes of symptoms who attended follow‐up rheumatology private and public clinics in Ibb City, the Republic of Yemen, for the period of September 1–November 30, 2021. About 20 healthy control volunteers matched for age and gender were enrolled in this study. Patients' diagnosis was carried out by rheumatology specialists based upon international clinical and laboratory criteria such as guidelines of the American Rheumatism Association and the European League Against Rheumatism 2010.^14^

A complete, filled‐out questionnaire was obtained for each patient. Questionnaire sociodemographic data includes the local habit of Qat chewing (a stimulant plant, i.e., chewed daily at afternoon, prevalent in Yemen and a few other West African countries). The rest of the data concerned disease history and fluctuation patterns such as the first date of RA diagnosis, symptom occurrence, and severity. It also includes some important data about symptoms such as joint symptoms (pain, swelling, warmth, and stiffness in the morning) and movement symptoms (difficulty walking, difficulty climbing stairs, and disability).

All participants provided informed consent to participate in the study. Ethical regulations, mainly the WMA Declaration of Helsinki—Ethical principles for medical research involving human subjects—2013, were met.

### 2.2 Laboratory analyses

Before patient treatment, about 5 ml of blood was collected for laboratory analyses, and healthy control volunteers were analyzed as well. A complete blood count (CBC) analysis was carried out on EDTA blood on an automatic hematology analyzer (Sysmex xsi‐500). A full CBC report was obtained, and the new inflammatory markers of leukocytes were calculated for each participant. The classical simple RA activity monitoring tests are also carried out on EDTA blood and serum samples, these tests are modified Westergren ESR, agglutination methods of CRP, and RF.

### 2.3 Statistical analysis

Statistical Package for Social Sciences (SPSS) version 19 was used for data analysis. Data were systematically analyzed with descriptive statistics such as mean, SD, range, frequency, and percentage as appropriate. Independent *t*‐test or Mann–Whitney rank‐sum Test was used to analyze the difference. The Spearman's correlation test was used for correlation analyses. Receiver operating characteristics (ROC) curve analysis was used to get the useful cutoff value of the new inflammatory markers upon Youden index calculation. A statistical test was considered significant at *p*‐value ≤ 0.05.

## 3 RESULTS

### 3.1 Sociodemographic and clinical data

The gender distribution of rheumatoid arthritis patients includes 77.4% females and 22.6% males, most of them aged 50 years and up (46.7%). The patients who chewed qat were 54.8%; meanwhile, the majority of patients (about 90.3%) didn't smoke (Table 1).

**Table 1:** Participant's sociodemographic data.

Data	No	Percentage
Sex		
Male	14	22.6
Female	48	77.4
Age groups		
Less than 20	3	4.9
20–29	8	13
30–39	11	17.7
40–49	11	17.7
50–60	18	29
More than 60	11	17.7
Smoking		
Yes	6	9.7
No	56	90.3
Qat chewing		
Yes	34	54.8
No	28	45.2

Table 2 summarizes the clinical data for rheumatoid arthritis patients. More than half of RA patients (53.2% had a disease duration of 2–5 years). Joint symptoms (pain, swelling, warmth, and stiffness) were prevalent among most patients (>85%). Thus, movement problems were exhibited by RA patients as difficulty walking, climbing stairs, and any form of disability, with a percentage of 98.3%, 80.6%, and 30.6%, respectively.

**Table 2:** Clinical data for rheumatoid arthritis patients.

Clinical data	No.	Percent
Duration (years)		
1	10	16.1
2–5	33	53.2
6–10	14	22.6
>10	5	8.1
Symptoms frequency		
Always	29	46.8
Intermittent	33	53.2
Joint symptoms		
Pain	61	98.3
Swelling	55	88.7
Warmth	53	85.4
Stiffness in the morning	53	85.4
Time of severe symptoms		
Morning	39	62.9
Evening	14	22.6
At sleep	16	25.8
At work	2	3.2
Movement symptoms		
Difficulty walking	61	98.3
Difficult climbing upstairs	50	80.6
Have disability	19	30.6

### 3.2 Laboratory analysis results

Laboratory investigations of RA patients and healthy controls showed a statistical difference in the majority of them, as shown in Table 3. All analysis results increased in RA patients except the mean monocyte count and hemoglobin level, which were lower than those of healthy volunteers. It was noted that both means of ESR and neutrophil to monocyte ratio (NMR) were similarly elevated by about four times in RA patients than in healthy, they were 40.13 ± 26.4 versus 10.3 ± 4.6 and 12.87 ± 15.47 versus 3.70 ± 2.58, respectively. The lymphocytes to monocytes ratio (LMR) also exhibited elevation in RA patients by about 2.6 times more than the healthy control (8.39 ± 11.44 vs. 3.16 ± 1.07).

**Table 3:** Mean laboratory parameters for rheumatoid arthritis patients and healthy volunteers.

Analysis	Patients Mean ± SD	Normal control Mean ± SD	*p*‐Value
Hb (g/dL)	12.9 ± 1.84	14.8 ± 1.11	0.001
WBCs (×10^9^/L)	7.83 ± 6.38	5.70 ± 1.66	0.019
PLTs (×10^9^/L)	311 ± 121.8	250 ± 56.2	0.003
PDW (fl)	14.2 ± 2.27	11.8 ± 1.08	0.001
N (×10^9^/L)	4.02 ± 2.96	2.41 ± 1.21	0.001
L (×10^9^/L)	2.49 ± 1.40	2.28 ± 0.71	0.367
M (×10^9^/L)	0.46 ± 0.24	0.79 ± 0.29	0.001
NMR	12.87 ± 15.47	3.70 ± 2.58	0.0001
LMR	8.39 ± 11.44	3.16 ± 1.07	0.001
NLR	1.86 ± 1.37	1.11 ± 0.59	0.001
ESR mm/h	40.13 ± 26.4	10.3 ± 4.6	0.0001
CRP titer IU	8.67 ± 6.79	1.45 ± 0.51	0.0001
RF titer IU	14.82 ± 5.63	1.80 ± 1.01	0.0001

Abbreviations: CRP, C‐reactive protein; ESR, erythrocyte sedimentation rate; Hb, hemoglobin; L, lymphocyte; LMR, monocyte–lymphocyte ratio; M, monocyte; N, neutrophil; NLR, neutrophil–lymphocyte ratio; NMR, neutrophil–monocyte ratio; PDW, platelet distribution width; PLTs, platelets; RF, rheumatoid factors; SD, standard deviation; WBCs, white blood cells.

### 3.3 Correlation between the new and classic inflammatory markers

Correlation studies reveal statistically significant weak and moderate correlations listed in Table 4. Total leukocyte count and platelet count were positively correlated with the classic markers (CRP, ESR, and RF). The absolute neutrophil count also positively correlated with these markers, which were stronger than total WBCs, in contrast, the absolute monocyte count negatively correlated with them. The new inflammatory markers NMR, LMR, and NLR were positively correlated with ESR (*r* = 0.529, *p* < 0.0001; *r* = 0.275, *p* = 0.012; *r* = 0.347, *p* = 0.002) respectively, and RF (*r* = 0.381, *p* < 0.0001; *r* = 0.300, *p* = 0.007; *r* = 0.227, *p* = 0.043), respectively. The correlation coefficient calculated between NMR and ESR was approximated by that calculated between ESR and RF (*r* = 0.529, *p* < 0.0001 and *r* = 0.517, *p* < 0.0001, respectively).

**Table 4:** Correlation between some CBC parameters and new inflammatory markers; NMR, LMR, and NLR ratios with the classic inflammatory markers among rheumatoid arthritis patients.

	CRP		ESR		RF	
	*r*	*p*	*r*	*p*	*r*	*p*
Hb	−0.284	0.010	−0.476	0.0001	−0.442	0.0001
WBCs	0.294	0.008	0.334	0.002	0.288	0.009
PLTs	0.339	0.002	0.287	0.009	0.245	0.027
N	0.278	0.013	0.441	0.0001	0.333	0.003
M			−0.321	0.003	−0.260	0.019
NMR			0.529	0.0001	0.381	0.0001
LMR			0.275	0.012	0.300	0.007
NLR			0.347	0.002	0.227	0.043
CRP			0.331	0.003	0.517	0.0001
RF			0.638	0.0001		

Abbreviations: CBC, complete blood count; CRP, C‐reactive protein; ESR, erythrocyte sedimentation rate; Hb, hemoglobin; LMR, monocyte–lymphocyte ratio; M, monocyte; N, neutrophil; NLR, neutrophil–lymphocyte ratio; NMR, neutrophil–monocyte ratio; PLTs, platelets; RF, rheumatoid factors; WBCs, white blood cells.

### 3.4 Evaluation of NMR, LMR, and NLR efficacy for RA using ROC curve analysis

**Figure 1:**
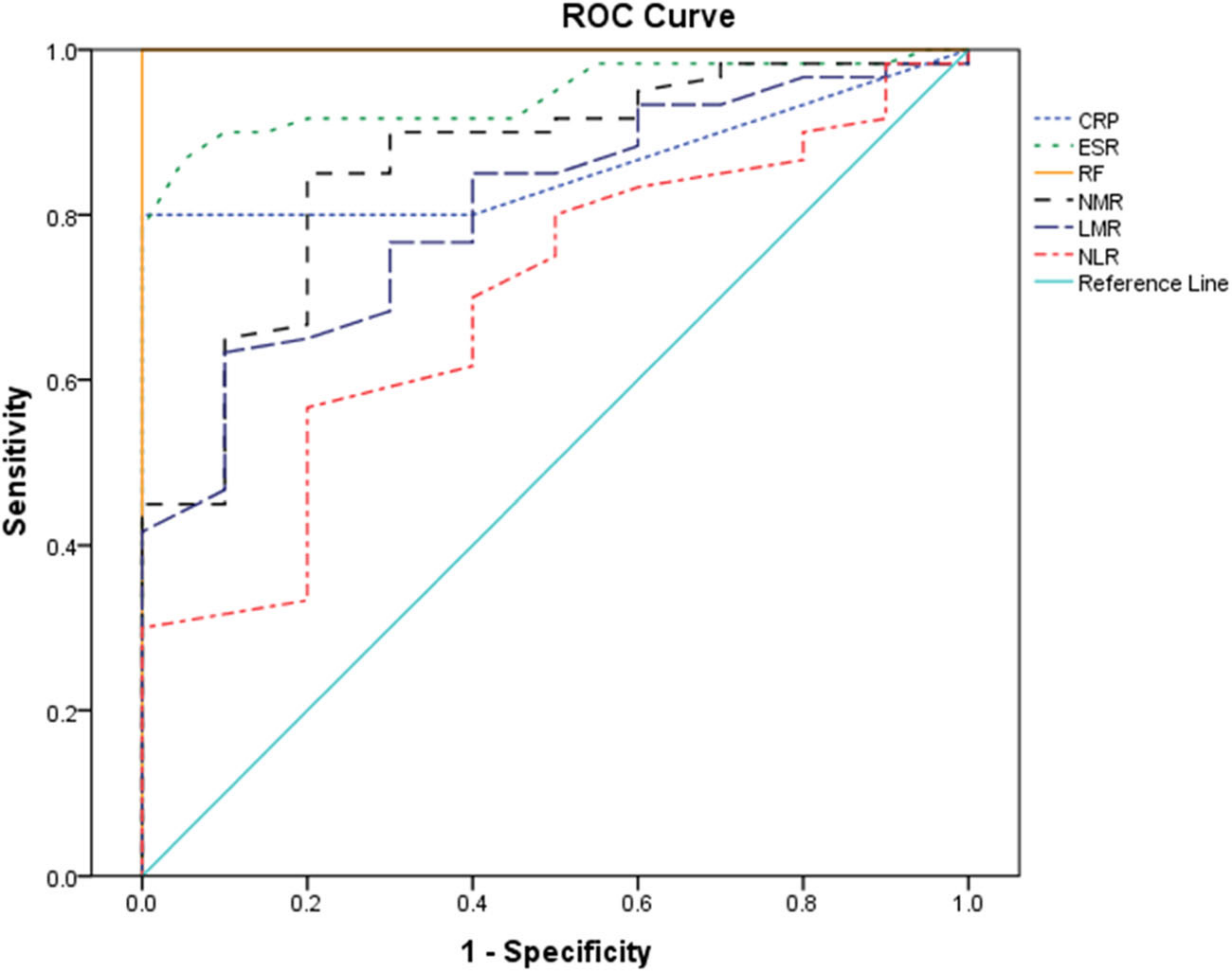
ROC curve of diagnostic biomarkers; NMR, LMR, NLR, CRP, ESR, and rheumatoid arthritis in Yemeni patients. CRP, C‐reactive protein; ESR, erythrocyte sedimentation rate; LMR, monocyte–lymphocyte ratio; NLR, neutrophil–lymphocyte ratio; NMR, neutrophil–monocyte ratio; RF, rheumatoid factors; ROC, receiver operating characteristic.

NMR cutoff value 4.7 gave an area under the curve (AUC = 0.861, 95% confidence interval [CI], 0.769–0.948) equal to that of CRP (AUC = 0.862, 95% CI, 0.780–0.940) and close to that of ESR (AUC = 0.945, 95% CI, 0.896–0.992). The sensitivity of the NMR was 85% higher than that of CRP (80%) and close to that of ESR (87%), but the specificity for them was 80%, 100%, and 95%, respectively. LMR and NLR cutoff values of 4.35 and 1.35 gave an (AUC = 0.807, 95% CI, 0.708–0.905) and (AUC = 0.699, 95% CI, 0.571–0.819) respectively, with high specificity and somewhat low sensitivity values (90%, 80%, 62%, and 57%, respectively). All details are depicted in Table 5 and Figure 1.

**Table 5:** Assessment of NMR, LMR, and NLR markers efficacy for RA follow‐up.

Marker	AUC (95% CI significance)	Cutoff	*p*	Sensitivity%	Specificity%
CRP IU	0.862 (0.780–0.940)	4	0.0001	80.3	100
ESR mm/L	0.945 (0.896–0.992)	19	0.0001	86.9	95
RF IU	1.000 (1.00–1.00)	5.5	0.0001	100	100
NMR	0.861 (0.769–0.948)	4.7	0.0001	85.2	80
LMR	0.807 (0.708–0.905)	4.35	0.0001	62.3	90
NLR	0.699 (0.571–0.819)	1.35	0.008	57.4	80

Abbreviations: AUC, area under the curve; CRP, C‐reactive protein; ESR, erythrocyte sedimentation rate; LMR, monocyte–lymphocyte ratio; NLR, neutrophil–lymphocyte ratio; NMR, neutrophil–monocyte ratio; RA, rheumatoid arthritis; RF, rheumatoid factors.

## 4 DISCUSSION

Rheumatoid arthritis is a common systemic autoimmune inflammatory disease. The inflammatory response causes synovitis in the joint, which leads to joint damage. Inflammatory process can establish the phase of the disease. In early RA, the involvement of antibodies in RA induction has been associated with a critical role for the IL‐23/Th17 axis in RA pathogenicity. At the late stage, RA may be characterized by altered cell death pathways in synoviocytes after long‐term exposure to inflammation.^15^ The systemic nature of this disease and involvement of innate immune cells (neutrophil and monocyte) in inflammation will both affect the proportion of these cells in peripheral blood and may create a certain distribution change of white blood cells, that is, a new balance, this change is assumed to be directly proportional to the RA disease activity. As a result, NMR, LMR, and NLR can be investigated as potential new inflammatory markers for RA activity.

All patients enrolled in this study attended rheumatology clinics while suffering from RA symptoms such as joint pain, swelling, warmth, stiffness, and movement problems as listed in Table 2. Thus, they were in the raised disease activity stage of the late phase, where 75.8% of patients had a disease duration of 2–10 years. In comparison with healthy control, there is an increase in inflammatory cells (total WBCs, neutrophils, lymphocytes, and platelets), as well as the classical RA markers (CRP, ESR, and RF), whereas monocytes decrease with a statistically significant difference from the control. When CRP, ESR, and RF are high, this suggests that the disease is very active.^16^ In parallel, the new inflammatory markers NMR, LMR, and NLR were elevated in accordance with previous studies.^11^, ^17^, ^18^, ^19^ Meanwhile, Lijuan et al.,^20^ showed that NLR and LMR may not be useful independent diagnostic or complementary markers for disease activity in RA patients. These hematological markers were positively correlated with the classical markers ESR and RF and, consequently, with RA disease activity. This association is very important because hematological markers are quite cheap and widely available.

It is worthy of mention that the NMR marker was an important one with the highest significance in all statistical analysis for difference and association. In addition, this marker exhibited the highest area under the curve value (AUC = 0.861, 95% CI, 0.769–0.948) with high diagnostic sensitivity (85%) and specificity (80%) among other markers close to that of CRP (AUC = 0.862, 95% CI, 0.780–0.940) and ESR (AUC = 0.945, 95% CI, 0.896–0.992). Spearman's correlation analysis showed that NMR had the highest correlation coefficient with ESR (*r* = 0.529, *p* < 0.0001) compared to the other markers. NMR represents two compartments; neutrophil that was directly correlated with ESR (*r* = 0.441, *p* < 0.0001), and the monocyte that was inversely correlated with ESR (*r* = −0.321, *p* = 0.003). The wonderful finding was that the mean NMR and ESR of RA patients were elevated four times more than those of the control group. All these results accentuate the diagnostic significance of the NMR marker for monitoring RA activity. The significance of NMR is attributed to the role of its components, neutrophils, and monocytes, in inflammation during RA symptom episodes. Neutrophils and monocytes are the main innate immune cells that modulate the course of inflammatory rheumatic diseases. Neutrophils are the primary‐acting cells, not monocytes.^21^ Our patients most likely lied at this stage. Monocytes appear after neutrophil infiltration according to the natural course of inflammation and secrete IL‐1β and TNF‐α, then IL‐17A is secreted from Th17 cells, these cytokines will amplify the inflammation.^22^

The usefulness of NMR as an inflammatory marker was reported previously. NMR is significantly associated with active ulcerative colitis disease—another inflammatory disease—and may be used in differentiating active from remission states.^23^ Elevated NMR was associated with poor prognosis in patients with pancreatic cancer.^24^ NMR can help in the identification of about 90% of COVID‐19 patients at high risk of ICU admission^25^; furthermore, NMR is an accurate predictor of in‐hospital mortality in the severe COVID‐19 patients.^26^

The first limitation of this study is the relatively small number of patients. This is due to the low prevalence of RA in Yemen and the limited health services in our poor country. The second limitation is the poor registry system among health providers. The third is the absence of public funding for research to increase the number of analyses.

The strength of this study starts with the nomination of one hematological marker (NMR) as a better one to be used in monitoring RA disease activity based upon appropriate statistical analysis. Then providing a cutoff value for clinical use. Finally, this study publishes data on Yemeni RF patients; to our best knowledge, the data for this population is scant.

## 5 CONCLUSION

The investigation of the new inflammatory markers obtained from routine hematology analysis—NMR, LMR, and NLR—as markers of RA activity in this study and many preceding research works indicated to their potential use in RA follow‐up to evaluate disease activity. This study searches for the best one by comparing their performances using the appropriate statistical analysis. Upon correlation studies and determination of the cutoff value and AUC for each marker, NMR was superior to LMR and NLR, while at the same time, its magnitude was very close to the classical markers CRP and ESR. Thus, NMR is a convenient and inexpensive inflammatory marker (with a value greater than 4.7) capable of displaying RA activity.

## AUTHOR CONTRIBUTIONS

**Jamil M A S Obaid**: Conceptualization; formal analysis; methodology; project administration; resources; supervision; writing—original draft. **Malikah M A Almjydy**: Investigation. **Maymouna A Q Garban**: Investigation. **Fatima S Q Al‐hebari**: Investigation. **Nusaibah A H Al‐washah**: Investigation.

## CONFLICT OF INTEREST STATEMENT

The authors declare no conflict interest.

## ETHICS STATEMENT

Informed consent for participation in the study was obtained from patients. This work met the international ethical guidelines, mainly the WMA Declaration of Helsinki—Ethical Principles for Medical Research Involving Human Subjects, 2013.

## TRANSPARENCY STATEMENT

The lead author Jamil M. A. S. Obaid affirms that this manuscript is an honest, accurate, and transparent account of the study being reported; that no important aspects of the study have been omitted; and that any discrepancies from the study as planned (and, if relevant, registered) have been explained.

## Data Availability

All data is available in this manuscript.
